# Does the Addition of Low-Dose Antibiotics Compromise the Mechanical Properties of Polymethylmethacrylate (PMMA)?

**DOI:** 10.3390/polym16162378

**Published:** 2024-08-22

**Authors:** Valentina Egger, Dietmar Dammerer, Gerald Degenhart, Johannes D. Pallua, Werner Schmölz, Martin Thaler, Klaus-Dieter Kühn, Michael Nogler, David Putzer

**Affiliations:** 1 Department of Orthopaedic and Traumatology, Medical University of Innsbruck, 6020 Innsbruck, Austria; valen.egger@gmail.com (V.E.); johannes.pallua@i-med.ac.at (J.D.P.); michael.nogler@i-med.ac.at (M.N.); 2 Department for Orthopaedics and Traumatology, University Hospital Krems, 3500 Krems an der Donau, Austria; dietmar.dammerer@krems.lknoe.at; 3 Department for Orthopedics and Traumatology, Karl Landsteiner University of Health Sciences, 3500 Krems an der Donau, Austria; 4 Department for Radiology, Medical University of Innsbruck, 6020 Innsbruck, Austria; gerald.degenhart@i-med.ac.at; 5 Helios Klinikum, Arthroplasty Center Munich West, 81241 Munich, Germany; martin.thaler@helios-gesundheit.de; 6 Center of Orthopaedics, Trauma Surgery and Rehabilitation Medicine, University of Greifswald, 17489 Greifswald, Germany; 7 Department for Orthopaedics and Traumatology, Medical University Graz, 8036 Graz, Austria; klaus.kuehn@medunigraz.at

**Keywords:** antibiotic-loaded bone cement, periprosthetic joint infection, local delivery of antibiotics, mechanical properties, gentamicin, compression testing

## Abstract

The increasing numbers of total joint replacements and related implant-associated infections demand solutions, which can provide a high-dose local delivery of antibiotics. Antibiotic-loaded bone cement (ALBC) is an accepted treatment method for infected joint arthroplasties. The mechanical properties of low-dose gentamicin-loaded bone cement (BC) in medium- and high-viscosity versions were compared to unloaded BC using a vacuum mixing system. As an additional control group, manual mixed unloaded BC was used. In a uniaxial compression test, ultimate compressive strength, compressive yield strength, and compression modulus of elasticity, as well as ultimate and yield strain, were determined according to ISO 5833-2022 guidelines. All groups exceeded the minimum compressive strength (70 MPa) specified in the ISO 5833 guidelines. Both ALBC groups showed a similar ultimate compressive and yield strength to the unloaded BC. The results showed that vacuum mixing increased the compression strength of BC. ALBC showed similar compressive strength to their non-antibiotic counterparts when vacuum mixing was performed. Added low-dose gentamicin acted as a plasticizer on bone cement. From a biomechanical point of view, the usage of gentamicin-based ALBC formulations is viable.

## 1. Introduction

Antibiotic-loaded bone cement (ALBC) is widely used in orthopedic surgery, especially in revision arthroplasty and periprosthetic joint infection (PJI) [1]. Demand for total joint replacements is high in current times but is expected to rise even further (by 174% for total knee arthroplasty and 673% for total hip arthroplasty) [2]. Despite great survival rates, the risk for PJI in primary joint arthroplasties is reported to range between 0.3% and 1.9%, and up to 10% in revision cases [3]. Two-thirds of PJI cases are caused through intraoperative inoculation of microorganisms, and the remaining infectious sources are hematogenous seeding and infection per continuitatem (after periprosthetic fracture, soft tissue infection, or osteomyelitis) [4]. Due to demographic transition, not only the number of people receiving joint arthroplasty is continuously rising, but also more patients have infection risk factors, such as obesity or diabetes [5]. Moreover, higher life expectancy causes longer dwelling times of prosthetic parts with an increased risk for hematogenous infection [6], while at the same time, sociocultural changes have led to higher expectations of mobility in the elderly population [2]. Hence, numbers of PJIs are expected to rise, and adequate treatment is more important than ever. Based on the rationale that high-dose local delivery of antibiotics is essential to eradicate biofilm-associated bacteria and the unavailability of a material that outranks acrylic bone cement in terms of performance and flexibility, ALBC remains the choice when it comes to septic/revision surgery [7,8]. ALBC can be categorized as either high- or low-dose ALBC (> 2 g < of antibiotic powder per 40 g batch of ALBC) [9]. High-dose ALBC is used in the first stage of musculoskeletal infection (MSKI) treatment (as a spacer), whereas low-dose ALBC is intended for use during primary arthroplasty as a prophylaxis in patients with risk factors (e.g., compromised tissue status, history of revisions), or in the second stage reconstruction for MSKI [10].

Revision surgery is a complex subject and requires an individual multi-disciplinary approach. Further research on risk factors requires distinct patient data and is therefore as important as it is difficult. Recently, contradictory studies about the efficacy of ALBC have been published [11,12]. Elusive inclusion criteria in retrospective studies, e.g., unregistered infection risk factors, as well as substantially lacking prospective clinical trials, may account for the inconsistent data on that subject [10].

Regardless of its efficacy in releasing antibiotics, the minimum requirement for ALBC is equal mechanical performance to native bone cement (BC) at a given balance of pharmaceutical benefits and risks. The ISO 5833:2002 guidelines provide both testing methods and values that the set cement should comply with. Biomechanical strength may be primarily quantified through compression and bending tests [13]. The following baseline properties are specified as a requirement in the ISO guidelines: average compressive strength ≥ 70 MPa, bending strength ≥ 50 MPa, and (elastic) bending modulus ≥ 1.8 GPa. The hypothesis that adding antibiotics alters the biomechanical properties of polymethylmethacrylate (PMMA) BC is supported by the following rationales [14]: First, antibiotic molecules act as radical scavengers, interfering with and inhibiting the formation of polymerization chains. Second, they may form clusters that become embedded within the PMMA matrix [15]. During antibiotic elusion, these clusters would dissolve, leaving voids in the cement. Consecutively, the increased porosity may impair the mechanical stability of the BC since every form of void can be a starting point of cement breakage [15]. Also, voids amplify the variability in strength-based measurements [16]. For safety reasons, it is crucial to ensure that ALBCs meet the ISO requirements for material strength. The biomechanical performance is not only affected by the addition of antibiotics [15,17,18,19,20,21,22,23,24] or changes in BC composition, but also by factors such as the cement’s viscosity [5,25], handling procedures, mixing method [26,27,28], and storage and preparation conditions, as well as the setting environment [29,30,31,32,33,34].

Comparison of study results is challenging when confounding factors are not specified in the materials and methods. To what extent the biomechanical properties are affected is still a matter of scientific discussion, and methodological precise research is crucial. In general, ALBCs are drug carriers with the aim of achieving the highest possible concentration of an antibiotic agent directly at the source of the infection (local delivery) without major systematic levels and the resulting side effects. Low-dose ALBC usually comes in commercially available compositions and in different viscosity versions [20] classified by the manufacturer. The representative antibiotic agent is gentamicin due to its broad-spectrum coverage [15]. While showing sufficient eradication ability towards Staphylococcus biofilms up until now, a noteworthy issue is the rise of resistant microbes as a consequence of natural selection [6,35]. With *Staphylococci* being the most frequent cause of PJI [36], other antibiotics such as tobramycin or combinations (e.g., with Clindamycin) try to solve this issue.

For gentamicin, Baleani found no difference in fatigue strength between hand-mixed low-viscosity Cemex RX and its commercially available 4.22% (*w*/*w*) gentamicin-infused counterpart [28]. Bitsch et al. discovered that the addition of 2 g gentamicin slightly decreased the compressive strength of manually mixed calcium-carbonate spacer cement, yet still followed the ISO guidelines well [21]. No initial effect was found on bending strength or bending modulus of elasticity [21]. Chen et al. experimented with manually mixed ALBC composition and its influence on porosity, antibiotic elution, and biomechanical stability [15]. A dose-dependent (0.01–0.4 g gentamicin) decrease in compression strength, with the 0.4 g gentamicin group falling just below the ISO threshold, was described by Chen et al. [15]. Also, Dunne et al. found that adding 1, 2, 3, and 4 g of gentamicin to Palacos RG led to a dose-dependent decrease in compressive and bending strength [23]. Vacuum-mixed BC containing more than 1.5 g gentamicin did not meet the ISO requirements [23]. Gálvez-Lòpez tested medium-viscosity BC with nine different types of antibiotics in high doses (4 g and 8 g antibiotic per 40 g batch) for elution and compressive strength with sample dimensions much bigger than recommended in the ISO [22]. Only the Rifampicin group was below the ISO requirements of 70 MPa; the other groups ranged between 76 and 586 MPa. Gentamicin-loaded BC was in the middle with 166 MPa, and the study’s extraordinarily high values have not been commented on [22]. These study results come to controversial conclusions about the effects on the biomechanical stability of ALBCs. As mentioned above, it is well known that the material strength of PMMA-BC is altered not only by its composition but also by handling modalities, working environment, and many more. Unfortunately, several studies fail to meet the required precision in methodology to compare them. Also, none of the mentioned studies addresses vacuum-mixed low-dose ALBC. Looking towards a future of rising numbers of total joint arthroplasties but also to a patient stock with higher risk factors simply due to longer life expectancies, the demand for prophylactic use of low-dose ALBC will increase, and reliable data on material strength is crucial.

The current study aimed to assess the mechanical stability of a vacuum-mixed low dose of gentamicin-loaded BC. We compared both the medium- and high-viscosity ALBC version to its likewise vacuum-mixed unloaded counterpart. Compression test results were quantified in ultimate compressive strength, compressive yield strength, and compression modulus of elasticity, as well as ultimate and yield strain. An additional hand-mixed control group was added to emphasize the influence of the mixing method.

## 2. Materials and Methods

### 2.1. Sample Preparation

We tested two commercially available ALBCs by Heraeus (Heraeus Medical GmbH, Hanau, Germany) varying in viscosity: high-viscosity Palacos^®^ R + G (group RG) [37] and medium-viscosity Palacos^®^ MV + G (group MVG) [38]. Both present levels of low-dose gentamicin of approximately 0.5 g (1.25%). Unloaded Palacos^®^ R (group R) [39] BC was used as a control in all experiments. The composition of the BCs is specified in Table 1. All cements were prepared according to their instructions using the corresponding vacuum mixing system Palamix^®^ (Heraeus Medical GmbH, Hanau, Germany). As an additional control group, Palacos R was also prepared by manual mixing under atmospheric conditions (group AR). Table 1 displays the characteristics of the study groups.

BC preparation was carried out by careful administration of the cement into a custom-built mold, so that test sample dimensions met the ISO 5833-2022 guidelines [16]. The target dimensions of the cylindrical test samples were 12 ± 1 mm in length and 6 ± 1 mm in diameter. Cautious attention was paid to complete filling and avoidance of air entrapment. Subsequently, two steel plates were mounted to seal the mold, fastened by four Allen screws with 15 Nm. After 30 min at room temperature (23 °C), the specimens were retrieved and underwent manual abrasive treatment with a saw to meet the ISO requirements. All samples were inspected for macroscopic defects and rested for another 24 h at room temperature for consolidation. Samples displaying substantial defects or dimensions beyond the range of tolerance were excluded. For each group, 10 samples underwent mechanical testing. The compressive strength was tested on cylindrical samples of BC 24 ± 2 h after molding and storage in dry air at room temperature (23 °C).

### 2.2. Micro-CT Analysis

To determine porosity of the prepared bone cement samples, a micro-CT analysis was performed using a vivaCT40 (Scanco Medical AG, Brüttisellen, Switzerland, 70 kV Xray tube voltage, 114 µA current, integration time of 600 ms, isometric voxelsize of 10.5 µm). Post-processing was performed using the Scanco Medical Software suite (Version 6.2 Scanco Medical AG, Brüttisellen, Switzerland). A cylindrical volume of interest (VOI) (diameter 675 voxels, 160 slices length) was selected in the center of the scanned object, and three different density threshold windows were chosen to represent visible differences in density distribution (550–1000 for the high-density particles, 220–550 as the mid-density matrix, 105–220 as the low-density matrix, and −999–105 as pores). The bone morphometric protocol was used to calculate the microstructural parameters of three different density windows. Parameters of interest were the mean particle diameter and the volume fraction between total volume and material selected within the threshold windows. The mean particle diameter was calculated by filling maximal spheres into a structure, and the average thickness of all voxels corresponded to the mean particle diameter.

Biomechanical testing was performed by a servo-hydraulic material testing machine (MTS, 858 MiniBionix II, Eden Prairie, MN, USA) with a 2.5 kN load cell along with a corresponding axial-torsional load transducer.

### 2.3. Uniaxial Compression Test

Compression testing was performed following the ISO 5833 guidelines [13]. The applied testing protocol defined a preload of 30 N and compressive loading with a constant axial speed of 20 mm per minute. Data recording was performed with 1000 Hz. The obtained data were transferred to MATLAB (Version 9.9, R2020b, The MathWorks, Inc., Natick, MA, USA) and GraphPad Prism (Version 8.0.1, GraphPad Software, Inc., La Jolla, CA, USA) to generate stress–strain curves. Cylindrical BC samples were compressed in the axial direction until failure occurred (2% yield point) as recommended by ISO 5833. The ultimate compressive strength σ_M_ (maximum applied load in N before specimen failure, real cross-sectional area in mm^2^ adjusted to the volumetric change), compressive yield strength σ_y_ (applied load in N at yield, real cross-sectional area in mm^2^ adjusted to the volumetric change), and elastic compression modulus in MPa (Young’s modulus, E, slope at 25–75% of maximum stress) were determined from the stress–strain curves using MATLAB (The MathWorks, Inc., Natick, MA, USA). Ultimate ε_M_ and yield strain ε_y_ were determined as the maximum of the first peak in the load strain curves.

### 2.4. Drug Elution Tests

Elution was determined at AZB (Analytisches Zentrum Biopharm, Berlin, Germany, accredited testing lab acc. to ISO 17025 [40]). BC samples (standardized cylindric specimens, diameter 25 mm, height 10 mm, surface 8.0 cm^2^) were stored at 37 °C in a 10.5 mL dissolution medium (0.1 M phosphate buffer, pH 7.4). Aliquots were taken, and the dissolution medium was renewed at the following sampling points: 1 h, 6 h, 12 h, 24 h, and 48 h. The dissolution medium samples were stored at −20 °C until analysis.

Ten calibration standards from 100 to 7500 ng/mL (for gentamicin), from 50 to 2500 ng/mL, from 25 to 25,000 ng/mL (for vancomycin), and from 25 to 10,000 ng/mL (for clindamycin), including a zero (blank without internal standard) as well as a blank sample (blank with internal standard), were prepared by spiking 200 µL of working solutions with corresponding internal standard working solutions (18 µL for gentamicin and tobramycin). These solutions were used for liquid chromatography with tandem mass spectrometry (LC-MS/MS) analysis.

The study samples were diluted by a factor of 20 for gentamicin and prepared according to the calibration standards by adding an internal standard working solution. Every sample was analyzed three times.

Concerning the LC-MS/MS conditions, chromatographic separation was performed on a modular HPLC 1200 Series (Agilent Technologies, Waldbronn, Germany) using a Luna C18 (II) column, 150 × 2 mm, with two C18, 4 × 2 mm, guard columns (Phenomenex, Aschaffenburg, Germany) thermostated at 25 °C. The injection volume was 2 µL. The mobile phase A was 0.11 M trifluoroacetic acid/methanol (50:50), and mobile phase B was acetonitrile. An isocratic separation was achieved with an A:B ratio of 95:5 at a flow rate of 0.25 mL/min. The run time was 2.5 min, and the total cycle time was less than 3 min.

Under the described conditions, the four gentamicin components C1, C2, C2a, and C1a co-eluted. The HPLC method was previously used by Heller et al. (2005) [41] to determine gentamicin in biopsy samples. The detection of the co-eluted gentamicin components was carried out using an API 4000 QTrap (Applied Biosystems, Darmstadt, Germany). Ionization was carried out with an electrospray interface (positive polarity) using the mass selective detector in the multiple reaction monitoring mode (MRM). The extracted ion chromatograms of the following ion transitions were stored and calculated: 478.4 → 322.3 *m*/*z* (gentamicin C1), 464.4 → 322.3 *m*/*z* (gentamicin C2 and C2a), 450.3 → 322.3 *m*/*z* (gentamicin C1a), and 468.4 → 163.1 *m*/*z* (internal standard). The three ion transitions of gentamicin components were summed by the software Analyst 1.4.2 (Applied Biosystems, Darmstadt, Germany).

The detection of tobramycin and the internal standard were carried out using an API 4000 QTrap (Applied Biosystems, Darmstadt, Germany). Ionization was carried out with an electrospray interface (positive polarity) using the mass selective detector in the multiple reaction monitoring mode (MRM). The extracted ion chromatograms of the following ion transitions were stored and calculated: 163.1 *m*/*z* (tobramycin) and 478.4 → 322.3 *m*/*z* (internal standard for tobramycin). The chromatograms were evaluated by the software Analyst 1.4.2 (Applied Biosystems, Darmstadt, Germany).

### 2.5. Statistics

Statistical analysis was performed with GraphPad Prism (Version 8.0.1, GraphPad Software, Inc., La Jolla, CA, USA). *p*-values *p* < 0.05 were considered statistically significant. The Kolmogorov–Smirnov test was carried out to assess normal distribution, and one-way ANOVA was used to compare study groups. All boxplot diagrams displayed values as means, standard deviation (SD), and minimum and maximum (min/max). The *t*-test was used to compare antibiotic elution rates for RG hand-mixed and RG vacuum-mixed.

Auxiliary symbols were used to visualize significance levels. They are to be interpreted as follows: * indicates 0.05 > *p* > 0.002, ** indicates 0.002 > *p* > 0.001, *** indicates *p* < 0.001.

Power analysis was performed using G*Power 3.1.9.7 (University of Kiel, Kiel, Germany). Post hoc calculated power analysis was performed using the given alpha 0.05, sample size n = 10, and effect size = 0.6 calculated from group parameters. The resulting power was 0.87.

## 3. Results

**Figure 1:**
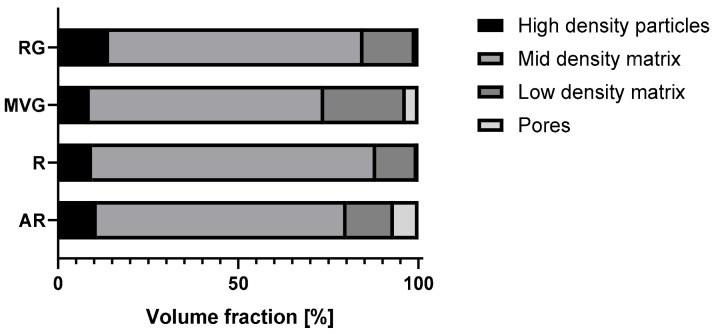
Distribution of the volume fraction in % across study groups.

Micro-CT analysis showed the particle size distribution within the application device used to produce the samples for mechanical testing. The results of the analysis are reported in Table 2, and the volume fraction composition of the samples is shown in Figure 1. RG showed the highest percentage of highest-density particles (14%), followed by AR (11%). In MVG and R, a similar percentage of highest-density particles (9%) was observed. AR showed the highest percentage of pores (7%) followed by MVG (3%). In MVG, the mean particle diameter of the pores was 0.2 mm, and for AR, it was 0.1 mm. In R and RG, the pore size was around 0.02 mm, which means a reduction by factor of 10 in comparison to the other two groups. A statistically significant higher mean particle size was found for MVG in comparison to R (*p* = 0.043) and RG (*p* = 0.039). The smallest percentage of pores was found for R (0.3%). The usage of a vacuum system could therefore reduce the presence of pores by a factor of 20. R showed also the most uniform distribution as it was composed mostly of mid-density and low-density matrix (90%). In RG, a shift towards the low-density matrix could be observed in comparison to R (R 11% and RG 14% low-density matrix). No statistically significant difference could be found between the groups for the low-density matrix (*p* = 0.523). MVG showed the highest percentage in the low-density matrix (23%) and the lowest mid-density matrix (56%). MVG showed the smallest mean particle diameter in the mid-density matrix. A statistically significant smaller mean particle diameter of MVG was observed in comparison to AR (*p* = 0.048) and R (*p* = 0.034). For the high-density particles, no statistically significant difference could be observed between the four groups under investigation (*p* = 0.333).

**Figure 2:**
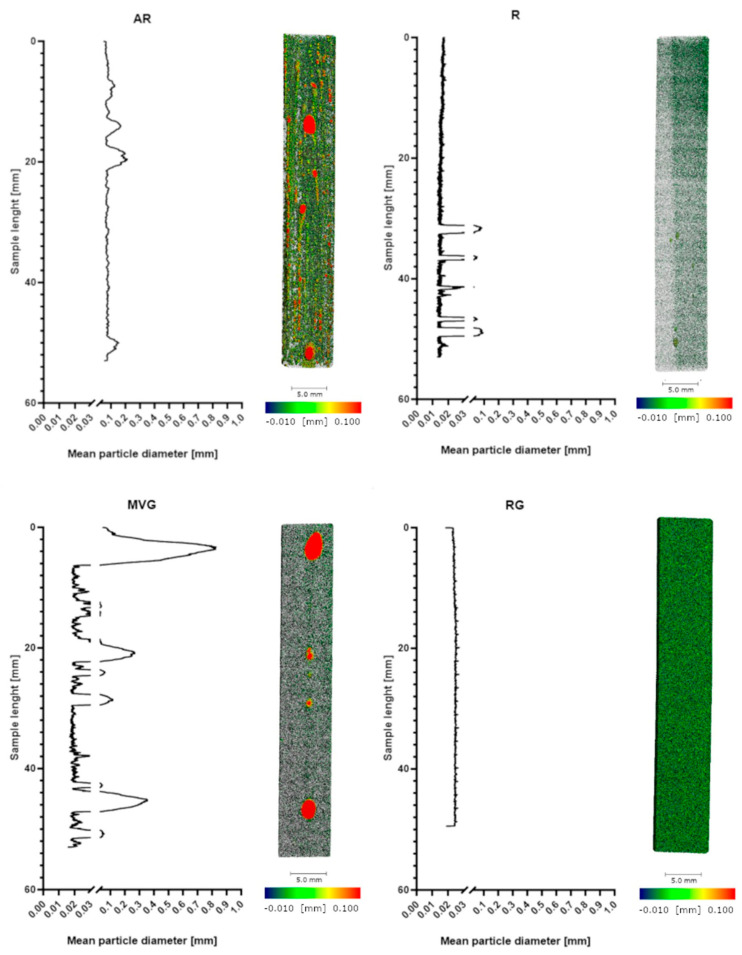
Micro-CT imaging in the 50% cross-section across study groups with color mapping indicating density level. Graphs show the mean particle diameter distribution along the samples for the four different groups under investigation.

In Figure 2, the distribution of the density along the samples of the four different study groups is shown. R and RG show a very uniform pattern with a close to uniform density. AR shows a less uniform pattern with some pores of varying sizes. In MVG, the density matrix is shown to be more uniform; however, there are several big pores present, although a vacuum system was used in the preparation of the samples. Also, in R, some pores could be found; however, they were less visible and smaller in diameter.

**Figure 3:**
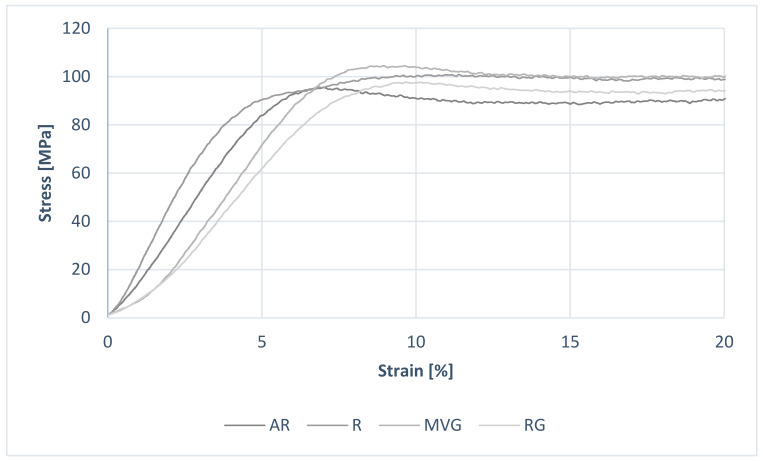
Example of stress–strain curves of the four different groups following uniaxial compression testing.

**Figure 4:**
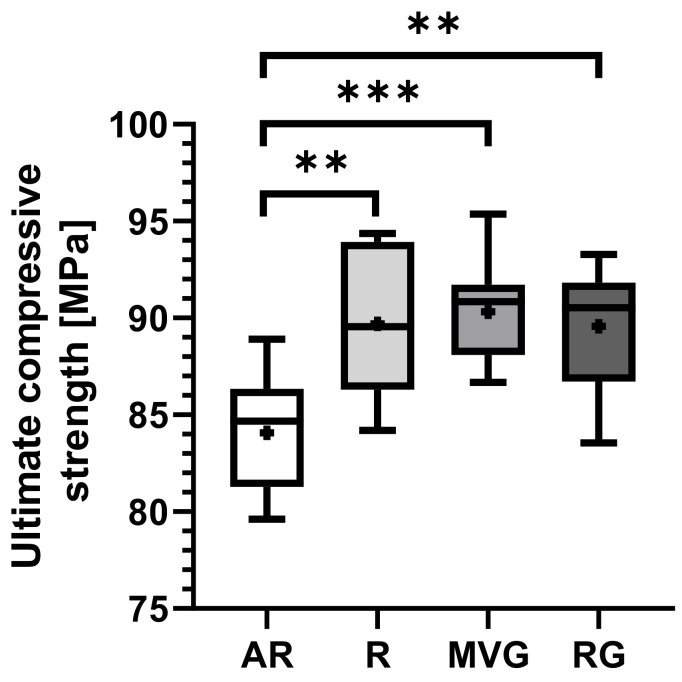
Box plots showing the mean ultimate compressive strength [MPa] as dots; the 25th, 50th, and 75th percentiles as boxes; and minimum/maximum as bars across study groups. ** indicates 0.002 > *p* > 0.001, *** indicates *p* < 0.001.

Considering the results from the uniaxial mechanical testing, all data within groups were normally distributed (*p* < 0.05). An example of the obtained stress–strain curves is shown in Figure 3. No statistically significant difference could be found between the R, MVG, and RG in terms of ultimate compressive strength (Figure 4, Table 3). MVG showed mean ultimate compressive strengths of 90.3 ± 2.6 MPa, R 89.7 ± 3.7 MPa, and RG 89.6 ± 3.2 MPa. AR showed a statistically significantly lower mean ultimate compressive strength of 84.1 ± 2.9 MPa in comparison to R (*p* = 0.002), MVG (*p* < 0.001), and RG (*p* = 0.002).

**Figure 5:**
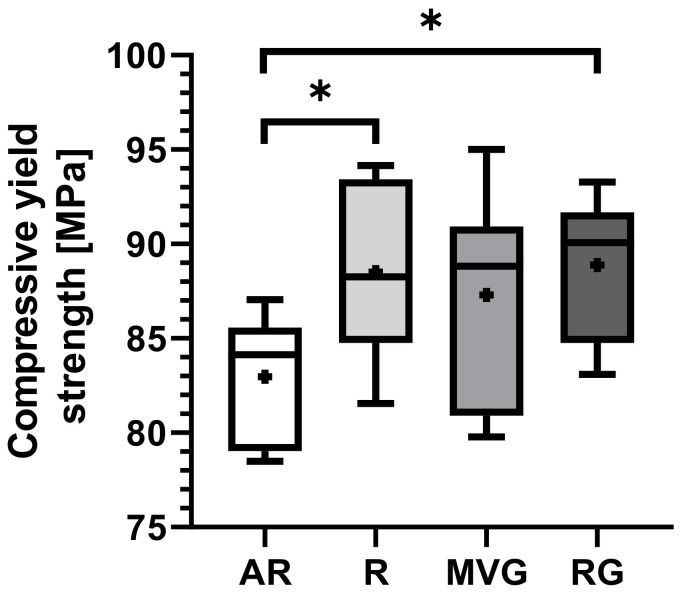
Box plots showing the mean yield strength [MPa] as dots; the 25th, 50th, and 75th percentiles as boxes; and minimum/maximum as bars across study groups. * indicates 0.05 > *p* > 0.002.

AR displays the lowest mean compressive yield strength of 83.0 ± 3.3 MPa, in comparison to the other groups R (88.5 ± 4.5 MPa), MVG (87.3 ± 5.1 MPa), and RG (88.9 ± 3.6 MPa) (Table 3). A statistically significant difference was found for AR in comparison to R (*p* = 0.027) and to RG (*p* = 0.017) (Figure 5). No statistically significant difference could be found between the other groups.

**Figure 6:**
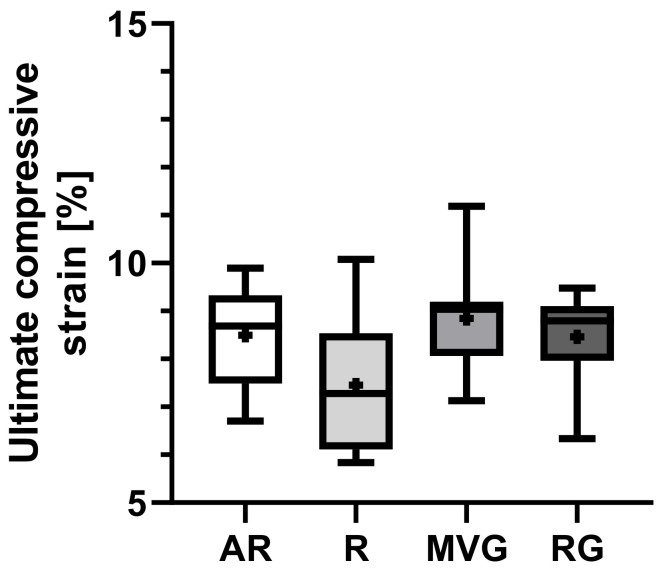
Box plots showing the mean ultimate compressive strain [%] as dots; the 25th, 50th, and 75th percentiles as boxes; and minimum/maximum as bars across study groups.

Group R presented a slightly lower mean ultimate compressive strain than the other groups (Figure 6), yet no statistically significant difference could be found (*p* = 0.694). The mean ultimate strain ranged from 7.4 ± 1.4% in R to 8.8 ± 1.1 in MVG (Table 2).

**Figure 7:**
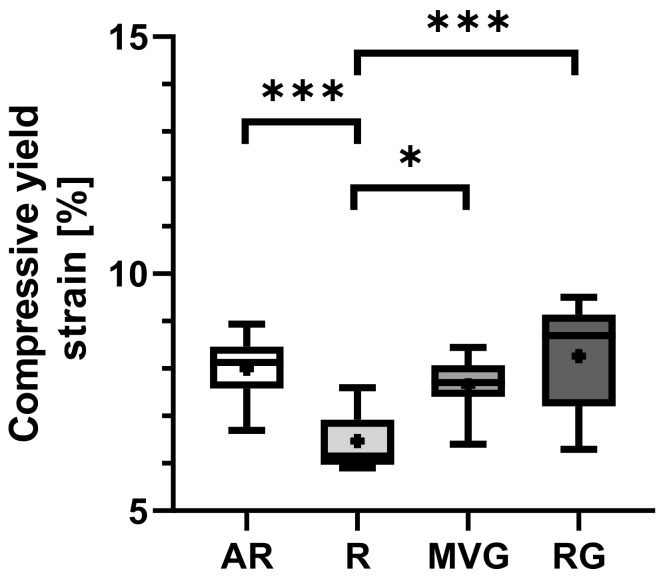
Box plots showing the mean yield strain [%] as dots; the 25th, 50th, and 75th percentiles as boxes; and minimum/maximum as bars across study groups. * indicates 0.05 > *p* > 0.002, *** indicates *p* < 0.001.

R showed a statistically significant lower mean compressive yield strain than AR (*p* < 0.001), MVG (*p* = 0.011), and RG (*p* < 0.001) (Figure 7), while no statistically significant difference between the other groups could be found (Table 3). R showed a mean compressive yield strain of 6.5 ± 0.6%, while AR, MVG, and RG were in the range of 8.4 to 8.8%.

**Figure 8:**
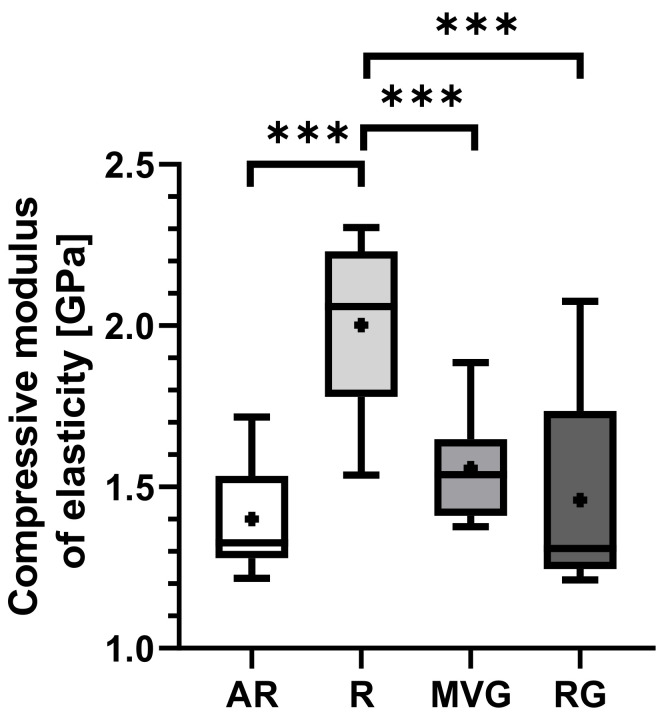
Box plots showing the mean compression modulus of elasticity [GPa] as dots; the 25th, 50th, and 75th percentiles as boxes; and minimum/maximum as bars across study groups. *** indicates *p* < 0.001.

Group R displayed the highest mean compression modulus of elasticity of 2.0 ± 0.3 GPa in comparison to AR with 1.4 ± 0.2 GPa (*p* < 0.001), MVG with 1.5 ± 0.2 GPa (*p* < 0.001), and RG 1.5 ± 0.3 GPa (*p* < 0.001) (Table 3). A statistically significant difference was found between R and all other groups (Figure 8), while all other pairwise comparisons showed no statistically significant difference.

**Figure 9:**
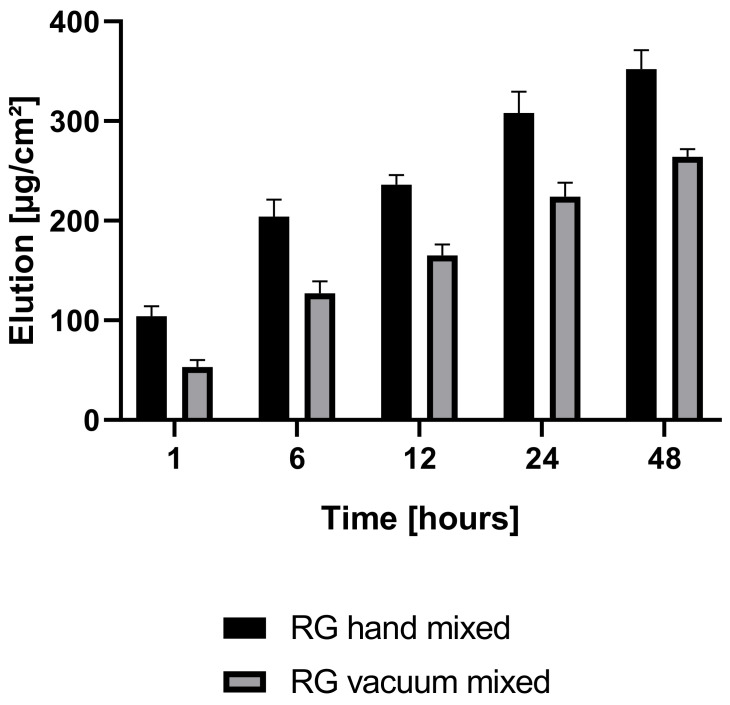
Antibiotic elution profile of hand-mixed and vacuum-mixed Palacos R + G cements.

Antibiotic elution profiles differed significantly between the two investigated groups, RG hand-mixed and RG vacuum-mixed (Figure 9). RG hand-mixed showed a statistically significant higher antibiotic elution profile than RG vacuum-mixed (1 h *p* = 0.002, 6 h *p* = 0.003, 12 h *p* = 0.001, 24 h *p* = 0.005, 48 h *p* = 0.002) in the first 48 h. The difference after 48 h in the drug elution rate was 25% between RG vacuum-mixed and RG hand-mixed. After one hour, the difference was 49% between the two groups—A higher release rate of antibiotics was obtained in the hand-mixed group, and the drug release occurred in a shorter period. Within one hour, 30% of the total drug elution recorded after 48 h was already released to the surrounding liquid, while in the vacuum-mixed group, only 20% was eluted.

## 4. Discussion

From the micro-CT analysis, RG showed the highest percentage of highest-density particles (14%), followed by AR (11%) and MVG and R (both 9%). AR showed the highest percentage of pores (7%), followed by MVG (3%). The high percentage of pores could be expected, as no vacuum system was used in the preparation. The MVG group, however, was prepared under vacuum conditions, although it was prepared according to the protocol and using the vacuum cartridge system of the manufacturer. It seems that the higher viscosity allowed some air bubbles to be trapped inside the bone cement matrix. In MVG, the mean particle diameter of the pores was 0.2 mm, and for AR, it was 0.1 mm. The air trapped was therefore creating bigger bubbles in the MVG group, as the viscosity of the material was higher. In R and RG, the pore size was around 0.02 mm, which means a reduction by a factor of 10 in comparison to the other two groups. The smallest percentage of pores was found for R (0.3%). The usage of a vacuum system could therefore reduce the presence of pores by a factor of 20. R also showed the most uniform distribution as it was composed mostly of mid-density and low-density matrices (90%). Adding antibiotics to the bone cement reduced the mid-density matrix and increased the percentage and low-density matrix. All four groups showed similar values for the high-density matrix. R and RG also showed a very uniform pattern with close to uniform density.

The possible presence of pores reduces the mechanical properties of the bone cement. In our study, all groups exceeded the minimum compressive strength (70 MPa) specified in the ISO 5833 guidelines. Both ALBC groups RG and MVG displayed the same ultimate compressive strength as group R (Figure 4), and the same trend was observed for yield strength (Figure 4). These findings suggest that low-dose gentamicin contained in commercially available BC does not impair its compressive strength at a given preparation method, which supports previous research conducted on that matter [23,26].

Also, our results corroborate that vacuum mixing increases the compression strength of BC (Figure 4). AR showed a highly significantly lower ultimate compressive strength than all vacuum-mixed groups under investigation. The same behavior was also observed for compressive yield strength. All vacuum-mixed groups displayed higher compressive yield strength than AR, although at lower significance levels. These observations are well in line with the findings of many different authors in the literature [20,26,27,28,42,43], who base the increased mechanical stability upon a reduction in air inclusion and consecutively in porosity. It seems furthermore that the viscosity has a minor influence on the compressive properties, since no difference could be found in any assessed measuring parameter between RG and MVG. The viscosity of BC has an impact on the penetration into the cancellous bone and therefore on the implant stability. BC of medium or high viscosity in the early application phase allows deeper penetration into the trabecular bone after removing the bone–fat–marrow debris [44,45,46]. High-viscosity cements, on the other hand, have a longer application window, allowing the surgeon more time for the correct alignment of the prosthesis. High-viscosity cement might be used for total knee arthroplasty procedures when the tibial and femoral implants are fixed with one cement batch.

Manually mixed BC shows air inclusions resulting in higher porosity, which results in lower mechanical properties, weakening the cement and promoting microfractures [29]. Vacuum mixing, on the other hand, provides a low-porosity cement paste, virtually without air inclusions [27], favorable mechanical properties, and therefore a lower risk of cement fracture [43]. Exhibiting a significant number of voids and pores is beneficial also for the creation of composite materials [47,48]. In our study, group R displayed lower values in strain and a significantly higher compression modulus of elasticity than the other groups, indicating a more rigid PMMA product. While our results were only significant for yield strain, the same trend could be seen in the ultimate strain at similar absolute numbers. Lower strain means lower deformation at the respective point of measurement, in our case at maximum load or yield. Similarly, higher values in compression modulus of elasticity signify higher rigidity of a material. RG shows a 25% increased yield strain and 25% lower compression modulus of elasticity compared to its non-antibiotic counterpart R, bringing it to a level of BC mixed under atmospheric conditions (AR). This leads to the assumption that even low doses of gentamicin have a plasticizing effect on highly viscous PMMA BC. Both ALBC groups RG and MVG displaying similar elastic properties to AR suggests that PMMA’s capacity to absorb energy (while keeping its structural integrity dimension-wise) decreases with the addition of low-dose gentamicin as it does after preparation under atmospheric condition mixing without vacuum.

This observation may be explained on a chemical/physical level:

Gentamicin, as a polar ionic compound, disorientates the elongating polymer chains, resulting in shorter chains, fewer atomic bonds, and weaker non-atomic bonds between the chains. This eventually creates a higher displacement of the molecules, meaning a more ductile material when subjected to load. The same behavior could be observed for MVG, although on a lower significance level in yield strain, implying that medium-viscosity cement may partially counteract the antibiotic-inducted increase in elasticity. Further investigations on medium-viscosity BC are needed to clarify this hypothesis.

Kühn reports similar levels of compressive strength for Palacos R (~80 MPa), Palacos RG (~88 MPa), and Palacos MVG (~90 MPa), which is well in line with our results (see Table 3) [25]. Compression modulus was not measured in this study. Bridges et al. reported an ultimate strength of 108 MPa for Palacos RG [49] following ISO 5833:2002 compression testing, which is higher than our results (90 MPa), but no information was provided for the curing/testing environment (temperature, wet/dry samples, days storage = extent of post-polymerization). Bishop et al. report a compressive yield strength of ~83 MPa for Palacos R and ~73 MPa for Palacos RG, while the compressive modulus was ~1.5 GPa for Palacos R and ~1.3 GPa for Palacos RG [50]. In our case, the determined values were higher than the ones reported in the study of Bishop et al. [50]; the water uptake during the 21 days of storage in BPS and biomechanical testing on wet samples may account for these differences. Kim et al. found a compression strength of ~83 MPa for wet Simplex P bone cement samples mixed with 0.5 g of Vancomycin and a compressive modulus of ~1.8 GPa [51] after three weeks of BPS storage. In this study, both compression strength and compression modulus did not significantly change with antibiotic loading below 2 g Vancomycin, whereas bending strength was compromised below ISO requirements already at 0.5 g Vancomycin added. Therefore, Vancomycin-loaded Simplex P is shown to have a higher compressive modulus than Palacos RG (1.5 GPa), while Palacos RG shows a higher compression strength (90 MPa) than Simplex P at equal Vancomycin concentrations.

Kühn et al. reported similar compressive strength levels for RG, MV, and MVG (~90 MPa), although plain R displayed a slightly lower average of ~80MPa [52]. Dunne et al. found no significant difference in compressive strength between Palacos R and Palacos RG. The overall slightly lower values (R 82 MPa, RG 79 MPa) may be explained by the pre-cooling of the BC [23].

PMMA BC shows a considerably lower strength when subjected to tensile loading than to compression or bending [53]. In a test carried out by Dunne, the tensile strength ranged between 32 and 56 MPa [23]. A strong correlation was seen between the ductility of the copolymers and the tensile strength [54]. Determined as the ratio of shear stress to shear strain, the shear strength of BC ranged between 32 and 63 MPa, depending on the method of cement preparation [23]. Implant–interface debonding has been known to initiate failure of cement femoral prothesis, and therefore shear strength is an important mechanical parameter. The interfacial static shear strength is highly influenced by surface roughness, and less by cement type and porosity of the BC [31]. Dunne found the fracture toughness to range between 1.21 and 1.85 MPa/m2 [23], and Lewis and Mladsie recognized a strong correlation between fracture toughness and impact strength [55]. One year earlier, Lewis stated that γ-irradiation produced a statistically significantly reduction in fracture toughness because of the related decrease in molecular weight [56]. Also, both the addition of radiopacifying agents or ABs and pores in the bone cement may have a destructive influence on the performance of the bone cement under sudden impact loads [31,57]. It has been postulated that creep of PMMA bone cement may be a causal factor in the loosening of cemented implant components. However, BC creep relaxes cement stresses and creates a more beneficial stress distribution at the interfaces [57]. Influencing factors on creep resistance are porosity, residual MMA monomer, powder particle size, fluid uptake, and the presence of copolymers or radio pacifiers, as well as test temperature and BC injection time [20]. Consequently, creep not only depends on the material properties, but also on the cement management in the surgical theater.

A significantly higher antibiotic elution was observed in hand-mixed RG when compared to vacuum-mixed RG. The same behavior can be expected also in the MVG group, although this was not investigated in this study. Reducing the voids in ALBC reduces porosity and therefore increases mechanical strength, although on the other hand, the antibiotic release rate is reduced. Antibiotic elution is said to compromise compression strength over time, since the released antibiotic leaves voids in the BC. Thus, the superficial porosity increases gradually, which might act as fracture initiation points or facilitate fracture propagation [58]. On the other hand, higher porosity also provides a higher surface area for antibiotic release. Given the mentioned low concentration of antibiotic and the low elution properties of ALBC [59], we consider this effect minor, yet additional studies are necessary to assess this. Also, a slightly (1–2%) increased porosity might facilitate osteointegration and can therefore be helpful [30]. ALBC hand-mixed cements might be used for temporary applications like as a spacer in a two-stage revision case, while ALBC vacuum-mixed might be used also for long-term applications. Even though this study followed the ISO requirements for material testing, correlations may be missed because of the small sample size. Furthermore, the unavailability of a non-antibiotic medium viscosity group left us only with data from other research groups as a reference. Already in this in vitro setting, the properties of BC change significantly with brand, composition, additives, mixing method, temperature, and storage conditions (duration, temperature, wet/dry). Precise methodology is crucial and often lacking to compare results. Finally, it should be remembered that analyzed parameters concern only a limited part of the biomechanical properties in an in vitro setting. A limitation of this study was that only PMMA from one manufacturer was analyzed. Conclusions drawn from the obtained results cannot be used to construct general recommendations, but they provide a good base for further research.

## 5. Conclusions

The study emphasizes the importance of precise methodology and considers how different preparation methods can influence the mechanical properties of PMMA bone cement in clinical applications. Hand-mixed bone cement (AR) exhibited higher porosity due to the absence of a vacuum system during preparation, which negatively affected its mechanical properties. Vacuum mixing significantly reduced porosity, leading to better mechanical strength, as evidenced by the superior performance of vacuum-mixed groups (R and RG) compared to manually mixed ones.

All bone cement groups surpassed the ISO 5833 guidelines for minimum compressive strength, with no adverse effects observed from the inclusion of low-dose gentamicin in commercially available BC. Gentamicin appeared to have a plasticizing effect on high-viscosity PMMA, slightly reducing the material’s rigidity.

The compressive strength of vacuum-mixed cements was higher than that of manually mixed ones, supporting the importance of reduced porosity. R displayed the highest rigidity, while the addition of gentamicin lowered the compression modulus, indicating a slight increase in ductility.

Reducing porosity via vacuum mixing improved mechanical strength but also reduced antibiotic elution, potentially impacting long-term performance. A balance between porosity and antibiotic release may be necessary, depending on the application, with hand-mixed ALBC potentially more suitable for temporary use and vacuum-mixed ALBC for long-term applications.

## Figures and Tables

**Table 1:** Main properties of the four different groups and preparation mode as specified by the manufacturer. The chemical compositions of the powder and liquids of Palacos^®^ R, RG, and MVG are reported. All values are given in rounded-off percentages. All three BCs also contain the coloring agent E141 and the radical scavenger hydroquinone.

Group	AR	R	MVG	RG
Commercial name	Palacos^®^ R [39]	Palacos^®^ R [39]	Palacos^®^ MV + G [38]	Palacos^®^ R + G [37]
Viscosity	high	high	medium	high
Vacuum mixing	-	+	+	+
Powder (g)/liquid (mL) ratio	40/20	40/20	44/20	40/20
Chemical composition powder [%]				
PMMA copolymer	84	84	87	84
Zirconium dioxide	15	15	12	15
Benzoyl peroxide	1	1	1	1
Gentamicin sulfate	-	-	1.25	1.25
Liquid composition [%]				
Methyl methacrylate	98	98	98	98
N,N-Dimethyl-p-toluidine	2	2	2	2

**Table 2:** Results of micro-CT analysis reporting the volume fraction and the mean particle diameter for different density thresholds.

	Thresholds	AR	R	MVG	RG	*p*-Value
Volume fraction	Pores	0.0675	0.0032	0.0349	0.0087	
	Low-density matrix	0.1315	0.1138	0.2264	0.1439	
	Mid-density matrix	0.6937	0.7887	0.6511	0.7054	
	High-density particles	0.1073	0.0944	0.0876	0.1419	
Mean particle diameter (SD)	Pores	0.094 (0.178)	0.022 (0.023)	0.283 (0.383)	0.018 (0.006)	0.027
	Low-density matrix	0.025 (0.008)	0.024 (0.007)	0.029 (0.009)	0.025 (0.008)	0.523
	Mid-density matrix	0.074 (0.017)	0.075 (0.016)	0.056 (0.013)	0.060 (0.013)	0.011
	High-density particles	0.032 (0.010)	0.036 (0.011)	0.036 (0.011)	0.041 (0.011)	0.333

**Table 3:** Results of compression strength (yield and ultimate), strain (yield and ultimate), and modulus of elasticity across study groups after compressive loading tests with 10 measurement repetitions. Values are given as mean (SD).

Group	Ultimate Strength σ_M_ [MPa]	Yield Strength σ_y_ [MPa]	Ultimate Strain ε_M_ [%]	Yield Strain ε_y_ [%]	Compression Modulus of Elasticity [GPa]
AR	84.1 (2.9)	83.0 (3.3)	8.5 (1.1)	7.9 (0.7)	1.4 (0.2)
R	89.7 (3.7)	88.5 (4.5)	7.4 (1.4)	6.5 (0.6)	2.0 (0.3)
MVG	90.3 (2.6)	87.3 (5.1)	8.8 (1.1)	7.6 (0.6)	1.6 (0.2)
RG	89.6 (3.2)	88.9 (3.6)	8.4 (0.9)	8.3 (1.1)	1.5 (0.3)
*p*-Value	<0.001	0.012	0.739	<0.001	<0.001

## Data Availability

Data will be provided on request due to legal reasons.
